# Dihydroartemisinin suppresses the susceptibility of *Anopheles stephensi* to *Plasmodium yoelii* by activating the Toll signaling pathway

**DOI:** 10.1186/s13071-024-06497-x

**Published:** 2024-10-04

**Authors:** Tingting Liu, Dan Zheng, Jing Wang, Xin Li, Shasha Yu, Zhilong Liu, Feifei Zheng, Caizhi Zhao, Xuesen Yang, Ying Wang

**Affiliations:** 1 Department of Tropical Medicine, College of Military Preventive Medicine, Army Medical University, No. 30 Gaotanyan St, Shapingba Dis, Chongqing, 400038 China; 2 School of Public Health, The Key Laboratory of Environmental Pollution Monitoring and Disease Control, Ministry of Education, Guizhou Medical University, Guiyang, 550025 China

**Keywords:** Dihydroartemisinin, *Anopheles stephensi*, *Plasmodium yoelii*, Susceptibility, Toll signaling pathway

## Abstract

**Background:**

Malaria is a serious public health concern. Artemisinin and its derivatives are first-line drugs for the treatment of *Plasmodium falciparum* malaria. In mammals, artemisinin exhibits potent anti-inflammatory and immunoregulatory properties. However, it is unclear whether artemisinin plays a regulatory role in the innate immunity of mosquitoes, thereby affecting the development of *Plasmodium* in *Anopheles* when artemisinin and its metabolites enter mosquitoes. This study aims to determine the effect of dihydroartemisinin (DHA), a first-generation semisynthetic derivative of artemisinin, on innate immunity and malaria vector competence of *Anopheles stephensi*.

**Methods:**

*Anopheles stephensi* was fed *Plasmodium*-infected mice treated with DHA via gavage, *Plasmodium*-infected blood containing DHA in vitro, or DHA-containing sugar, followed by *Plasmodium yoelii* infection. The engorged female mosquitoes were separated and dissected 8 and 17 days after infection. *Plasmodium* oocysts and sporozoites were counted and compared between the control and DHA-treated groups. Additionally, total RNA and proteins were extracted from engorged mosquitoes 24 and 72 h post infection (hpi). Real-time polymerase chain reaction (PCR) and western blotting were performed to detect the transcriptional levels and protein expression of immune molecules in mosquitoes. Finally, the Toll signaling pathway was inhibited via RNA interference and the infection density was analyzed to confirm the role of the Toll signaling pathway in the effect of DHA on the vector competence of mosquitoes.

**Results:**

DHA treatment via different approaches significantly reduced the number of *Plasmodium* oocysts and sporozoites in mosquitoes. The transcriptional levels of anti-*Plasmodium* immune genes (including *TEP1*, *LRIM1*, and *APL1C*), Toll pathway genes (including *Tube*, *MyD88*, and *Rel1*), and the effector *defensin 1* were upregulated by DHA treatment at 24 and 72 hpi. TEP1 and Rel1 protein expression was significantly induced under DHA treatment. However, Rel1 knockdown in DHA-treated mosquitoes abrogated DHA-mediated refractoriness to *Plasmodium* infection.

**Conclusions:**

DHA treatment effectively inhibited the development of *P. yoelii* in *A. stephensi* by upregulating mosquitoes’ Toll signaling pathway, thereby influencing the susceptibility of *Anopheles* to *Plasmodium*.

**Graphical Abstract:**

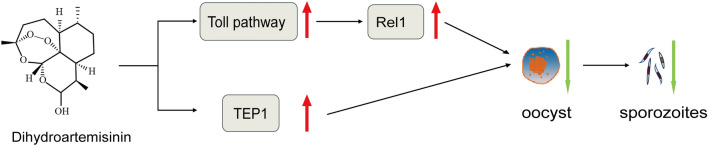

**Supplementary Information:**

The online version contains supplementary material available at 10.1186/s13071-024-06497-x.

## Background

Malaria is a serious public health concern caused by *Plasmodium* infection and is mainly transmitted by *Anopheles* mosquitoes. According to the latest World Malaria Report released by the World Health Organization (WHO), there were an estimated 249 million malaria cases and 608,000 deaths in 2022 [1]. In 2006, artemisinin-based combination therapies (ACTs) were recommended by the WHO as the first-line standard treatment for *Plasmodium falciparum* malaria [2]. The WHO believes that ACTs is the most effective treatment for malaria and the most effective drug for combating drug resistance [3]. Artemisinin has saved millions of lives, especially in malaria-affected Africa. According to WHO statistics, about 240 million people in sub-Saharan Africa have benefited from artemisinin combination therapies since 2000, and about 1.5 million malaria-related deaths have been averted due to ACTs [4].

Artemisinin and its derivatives reduce the number of malaria parasites and relieves malaria symptoms by interfering with the life cycle of the erythorcytic stage of the malaria parasite, especially affecting the formation of merozoites in red blood cells. Artemisinin comprises a group of sesquiterpene trioxane lactones extracted from the Chinese herb *Artemisia annua* L. [5]. Dihydroartemisinin (DHA) is a first-generation semisynthetic derivative of artemisinin. DHA is a sesquiterpene endoperoxide with prominent antimalarial efficacy that was discovered by Youyou Tu through the C-10 reduction of lactone carbonyl by sodium borohydride in the 1970s [6, 7]. The peroxy group is critical for the antimalarial activity. Functional groups interact with iron ions to produce highly reactive oxygen radicals, which can act on proteins, nucleic acids, and lipids of malaria parasites [8]. Artemisinins kill parasites by interfering with their surface membrane and mitochondrial function of the malaria parasite, ‌thereby blocking nutrients provided to the parasite by host red blood cells.

Artemisinin and its derivatives are the first-choice antimalarial drugs worldwide [9]. A campaign for artemisinin/DHA-piperaquine administration was conducted in malaria-endemic areas and was verified as safe and effective in improving malaria protection and decreasing malaria incidence [10, 11]. In addition to malaria treatment, artemisinin and its derivatives are potentially effective drugs for treating various helminthic diseases [12]. Besides, artemisinin and its derivatives have anti-inflammatory and immunoregulatory activities [13–15]. Artemisinin can shift the immune responses from T-helper 1 (T_H_1) to T-helper 2 (T_H_2), which is helpful in treatment of autoimmune encephalomyelitis [16]. Artemisitene significantly attenuated inflammatory response in DSS-induced ulcerative colitis via regulating the production of reactive oxygen species (ROS) [17]. Artemisinin inhibits accumulation and function of myeloid-derived suppressor cells via PI3K/AKT, mTOR, and MAPK pathways and enhances anti-PD-L1 immunotherapy in melanoma and liver tumors [18]. Artemisinin can regulate signaling pathways, such as NF-κB and NO synthase (NOS), and enrich ROS by binding with iron and NF-κB [19, 20]. As known, NOS and ROS also play an important role in the innate immune response of mosquitoes. However, these studies were limited to mammals, and it is unclear whether artemisinin plays a role in regulating mosquito immunity. When mosquitoes bite the blood of patients treated with artemisinin, artemisinin and its metabolites may enter the mosquitoes and affect their biological characteristics, such as their innate immune response to pathogens, which may further affect the development of malaria parasites in mosquitoes and influence the vector competence of *Anopheles* to *Plasmodium*.

This study aims to determine the effect of DHA on innate immunity and malaria vector competence of *Anopheles stephensi*. This study provides a theoretical basis for the development of new agents that can block malarial transmission by modulating the immune response of *Anopheles* mosquitoes.

## Methods

### Mosquito rearing and infection

The *A. stephensi* Hor strain was maintained at 28 ℃ and 70–80% relative humidity with a 12 h light–dark photocycle, according to the standard rearing procedures in the laboratory. The red fluorescent protein-transgenic *P. yoelii* BY265 strain was recovered and passed through the mice. The, 3–5-day-old female *A. stephensi* mosquitoes fed on *P. yoelii*-infected Kunming mice aged 4–6 weeks with 5–10% parasitemia at 24 ℃, as described previously [21]. The mosquitoes were anesthetized with carbon dioxide and dissected 8 and 17 days after infection. *Plasmodium* oocysts and sporozoites were counted under a fluorescence microscope. The infection rate and density were analyzed statistically.

### DHA treatment

DHA was purchased from Shanghai Macklin Biochemical Technology and dissolved in dimethyl sulfoxide (DMSO) to 100 mM as stock solution. The following three different DHA treatment methods were performed: (1) *Plasmodium*-infected mice were randomly divided into two groups, the control group and the DHA group, prior to blood feeding by the mosquitoes. The mice of the DHA group were orally administered 30 mg/kg of DHA (diluted with physiological saline) via gavage [22, 23]. Control mice were administered equal volumes of physiological saline containing the same amount of DMSO via oral gavage [24]. (2) The blood of *Plasmodium*-infected mice was collected and divided into two equal parts, followed by the addition of DHA to a final working concentration (50 μM) or equal volume of DMSO. The two groups of mosquitoes were fed artificial membrane blood in parallel in vitro through a rubber tube connected to a membrane blood infection system. The temperature of the blood sample was maintained at 37 ℃ using the flowing water action of the constant temperature water bath system [25]. (3) The DHA stock solution was diluted using 10% sugar solution to a final working concentration (50 μM). The 3–5-day-old female *A. stephensi* were fed the DHA-containing sugar solution for three consecutive days prior to infection with *P. yoelii*. Age-matched mosquitoes fed a sugar solution containing the same concentration of DMSO alone were used as controls.

### RNA isolation, complementary DNA (cDNA) synthesis, and quantitative PCR (qPCR)

Ten female *A. stephensi* mosquitoes from each group were anesthetized with CO_2_ and used for gene transcript analysis 24 and 72 h postblood feeding. Total RNA was extracted in accordance with the instruction of the HiPure Universal RNA Mini Kit (Magen, Guangzhou, Guangdong, China) and reverse transcribed to cDNA using the Reverse Transcription Kit (Takara, Dalian, Liaoning, China). Then, real-time quantitative PCR was performed using the KAPA SYBR^®^ FAST qPCR Kit (KAPA Biosystems, Wilmington, MA) with a Bio-Rad CFX96 Touch™ Real-time PCR instrument (Bio-Rad, Hercules, CA) to determine the transcriptional levels of immune genes, such as *TEP1, APL1, LRIM1, MyD88, Tube, Rel1, Cactus*, and*defensin 1* (*DEF1*), using the conserved S7 as the internal reference gene. The primers used are listed in Supplementary Table 1. The expression of each gene relative to the ribosomal S7 RNA was determined using the 2^−ΔΔCT^ method.

### Western blot

Fifteen *A. stephensi* mosquitoes from each group were homogenized in RIPA lysis buffer [50 mM Tris (pH 7.4), 150 mM NaCl, 1% sodium deoxycholate, 1% Triton X-100, 1 mM EDTA, 0.1% sodium dodecyl sulfate (SDS), 1× protease inhibitor, and 1 × phosphatase inhibitor] at 24 and 72 h postblood meal. Total protein was extracted and denatured at 95 °C for 5 min. Then the protein samples were separated using 10% SDS–polyacrylamide gel electrophoresis (PAGE) and transferred to a PVDF membrane for 1 h at 100 V. The membrane was blocked in 5% skimmed milk powder for 1 h at room temperature and incubated with primary antibodies against TEP1, Rel1, and the reference protein β-actin at 4 ℃ overnight. The membranes were washed three times with 1× TBST for 5 min and then incubated with an antirabbit secondary antibody for 1 h at room temperature. After washing, the membrane was visualized using the Chemi DOCTM MP Imaging System (BIO-RAD). ImageJ software was used to quantify signal intensity.

### RNA interference

RNA interference (RNAi) was conducted to further confirm the role of the Toll signaling pathway in the effect of DHA treatment on susceptibility of *A. stephensi* to *P. yoelii*. To synthesize the double-stranded RNA (dsRNA) of Rel1, *A. stephensi* cDNA was subjected PCR using gene-specific primers with a 5′ extension of T7 promoter tags (5′-TAATACGACTCACTATAGGG-3′) (Supplementary Table 1). The GFP fragment was used as a control dsRNA. The PCR products were used as templates for in vitro transcription reactions using the MEGAscript T7 Kit (Ambion Life Technologies, Austin, TX), following the manufacturer’s instructions. Then 3–5-day-old female mosquitoes were intrathoracically injected with 69 nl dsRel1 using a Nanoject II microinjector (Drummond Scientific Co., Bromall, PA). Equal amounts of double-stranded GFP (dsGFP) were used as a control. Gene silencing efficiency was detected 3 days after dsRNA injection by real-time quantitative PCR, as described above. The mosquitoes were challenged with *P. yoelii* BY265RFP via blood feeding. Mosquitoes were dissected and the oocysts in the midguts were counted under a fluorescence microscope 8 days after infection. The infection rates and densities were calculated.

## Statistical analysis

All statistical analyses were performed using GraphPad Prism (version 8.0) and SigmaStat (version 3.5) software. The infection rates of *A. stephensi* were analyzed using the chi-square test. A Student’s *t*-test was used to compare normally distributed data, and the Mann–Whitney *U*-test was used for non-normally distributed data. Statistical significance was set at *P* < 0.05.

## Result

### DHA gavage in mice decreased *P. yoelii* infection in *A. stephensi*

**Fig. 1:**
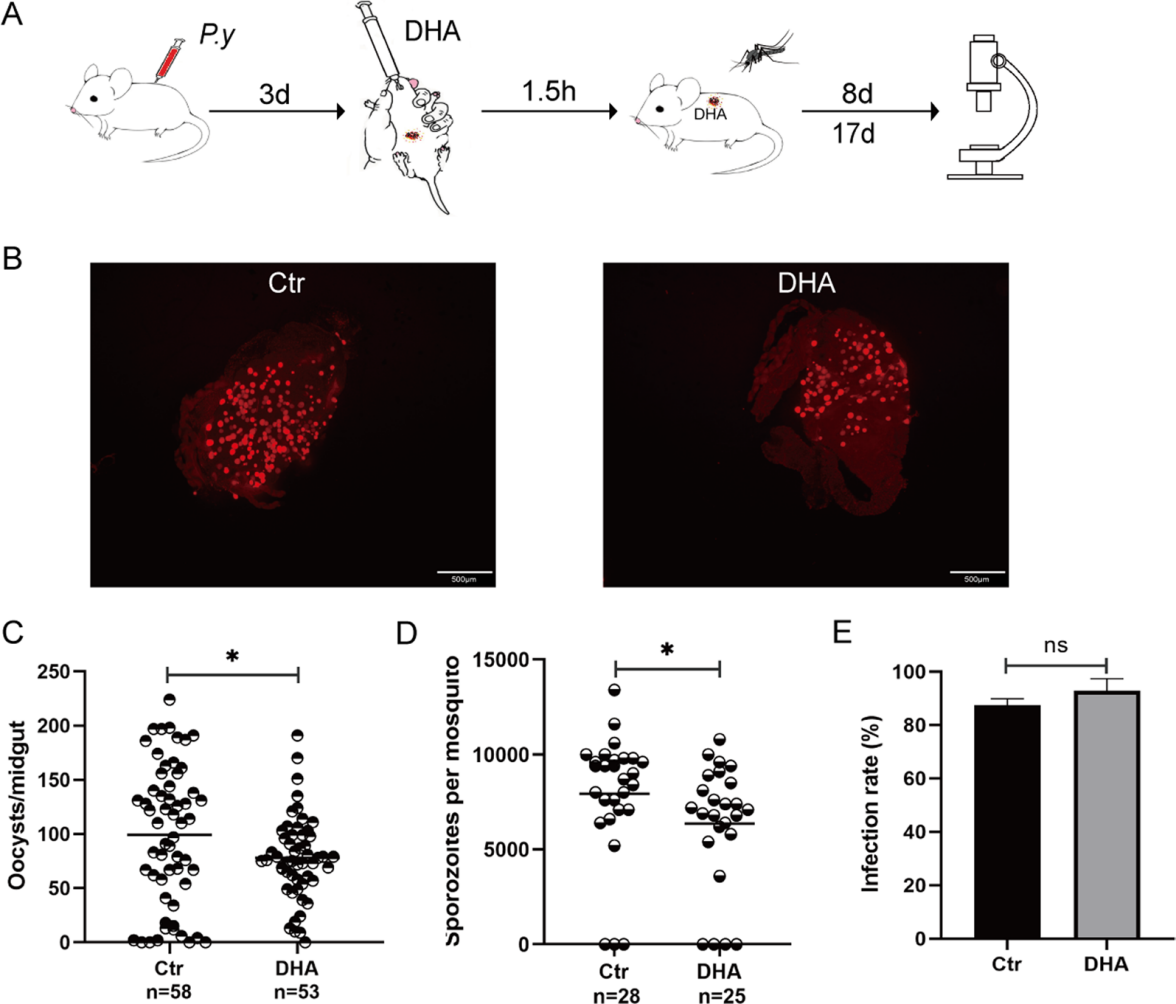
Feeding on DHA-gavage mice influenced *P. yoelii* infection in *A. stephensi*. **A** Schematic overview of DHA treatment and *A. stephensi* infection by *P. yoelii*. Vehicle solution-treated mice were used as controls. **B** The oocysts of *P. yoelii* in *A. stephensi*. **C**, **D** Oocyst and sporozoite counts in mosquitoes feeding on the DHA-gavage and control mice. **E** Comparison of infection rates of oocysts. Significance was determined by Mann–Whitney tests in **C** and **D** and chi-square test in **E**; *P < 0.05

To understand whether taking blood from malaria patients treated with artemisinin drugs affects the development of malaria parasites in *Anopheles*, *Plasmodium*-infected mice were orally administered DHA or DMSO to physiological saline via gavage. *Anopheles* were allowed to feed on DHA or control mice 1.5 h after gavage. Oocysts and sporozoites were counted 8 and 17 days after infection respectively (Fig. 1A). The results showed that mosquitoes fed on DHA mice via gavage had substantially less oocysts and sporozoites than mosquitoes fed on control mice (*P* < 0.05) (Fig. 1B–D). No significant difference was found in infection rates (*χ*^2^ = 2.456, *df* = 1, *P* = 0.117) (Fig. 1E). These results suggest that taking blood from DHA-treated mice inhibited *Plasmodium* development in *A. stephensi*, indicating a reduced susceptibility of *Anopheles* to *Plasmodium*.

### Blood-feeding in vitro with DHA inhibited *P. yoelii* infection in *A. stephensi*

**Fig. 2:**
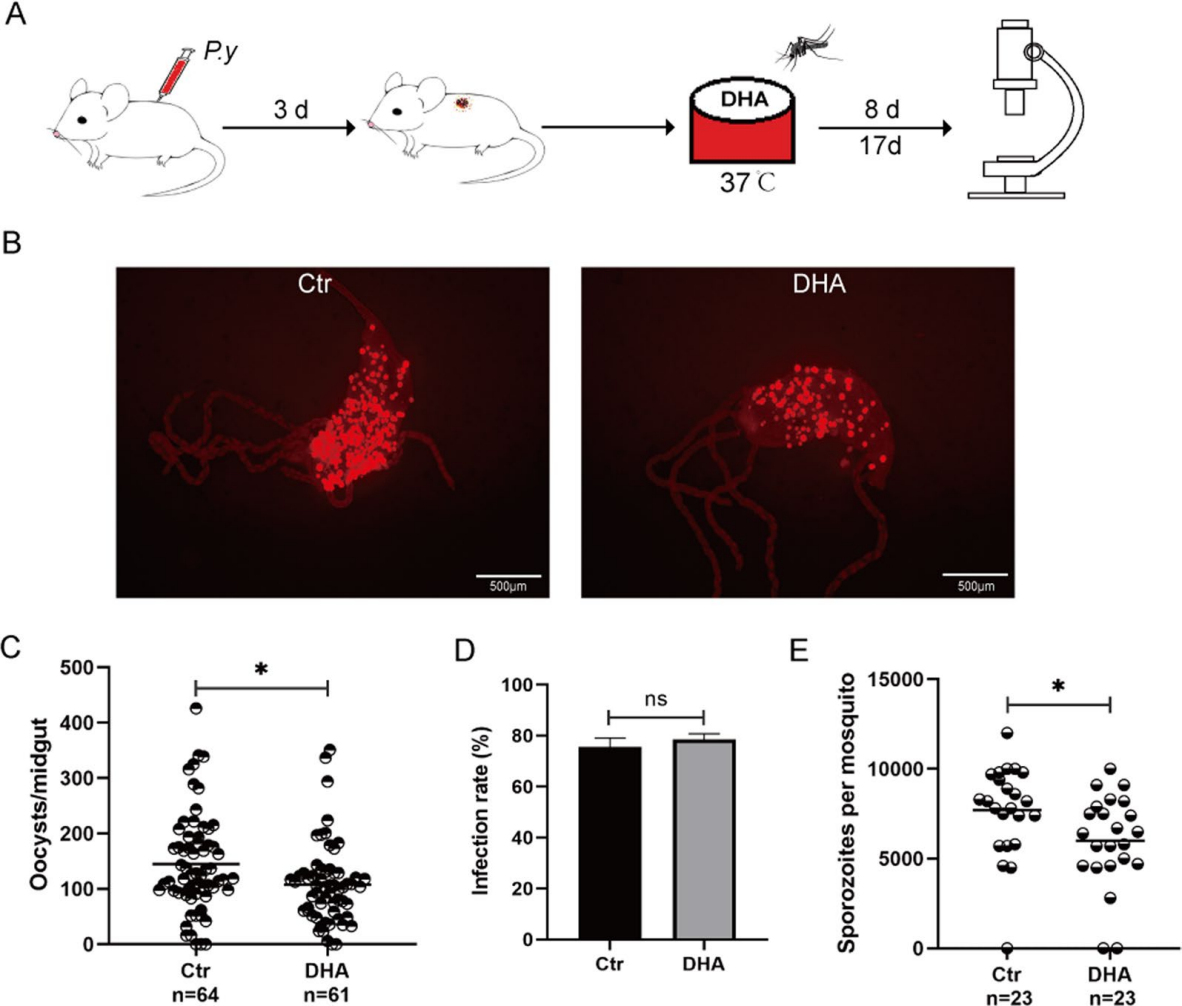
Blood-feeding in vitro with DHA inhibited *P. yoelii* infection in *A. stephensi*. **A** Schematic overview of DHA treatment and *A. stephensi* infection by *P. yoelii*. **B** The oocysts of *P. yoelii* in *A. stephensi*. **C** Oocyst counts in mosquitoes after blood-feeding in vitro with DHA or control reagent. **D** Comparison of infection rates of oocysts. **E** Sporozoite counts in mosquitoes after blood-feeding in vitro with DHA or control reagent. Significance was determined by Mann–Whitney tests in **C** and **E** and chi-square test in **D**; *P < 0.05

To exclude the effect of DHA on gametocytes in mice, which might affect the development of parasites in mosquitoes, a blood feeding in vitro method was used. The blood of *P. yoelii*-infected mice was obtained and added with DHA to a final concentration of 50 μM. The mosquitoes were then artificially blood-fed and the oocysts and sporozoites were counted (Fig. 2A). The comparison of infection densities and rates showed that the oocyst counts in the DHA group were significantly lower than those in the control group (*P* < 0.05) (Fig. 2B, C), and there was no significant difference in infection rates (*χ*^2^ = 0.149, *df* = 1, *P* = 0.699) (Fig. 2D). The numbers of sporozoites in DHA treatment group were significantly lower than those in the control group (*P* < 0.05) (Fig. 2E). This indicates that in vitro blood feeding with DHA inhibited the development of oocysts and sporozoites in *A. stephensi*.

### Feeding *A. stephensi* with DHA containing sugar inhibited *P. yoelii* oocyst development

**Fig. 3:**
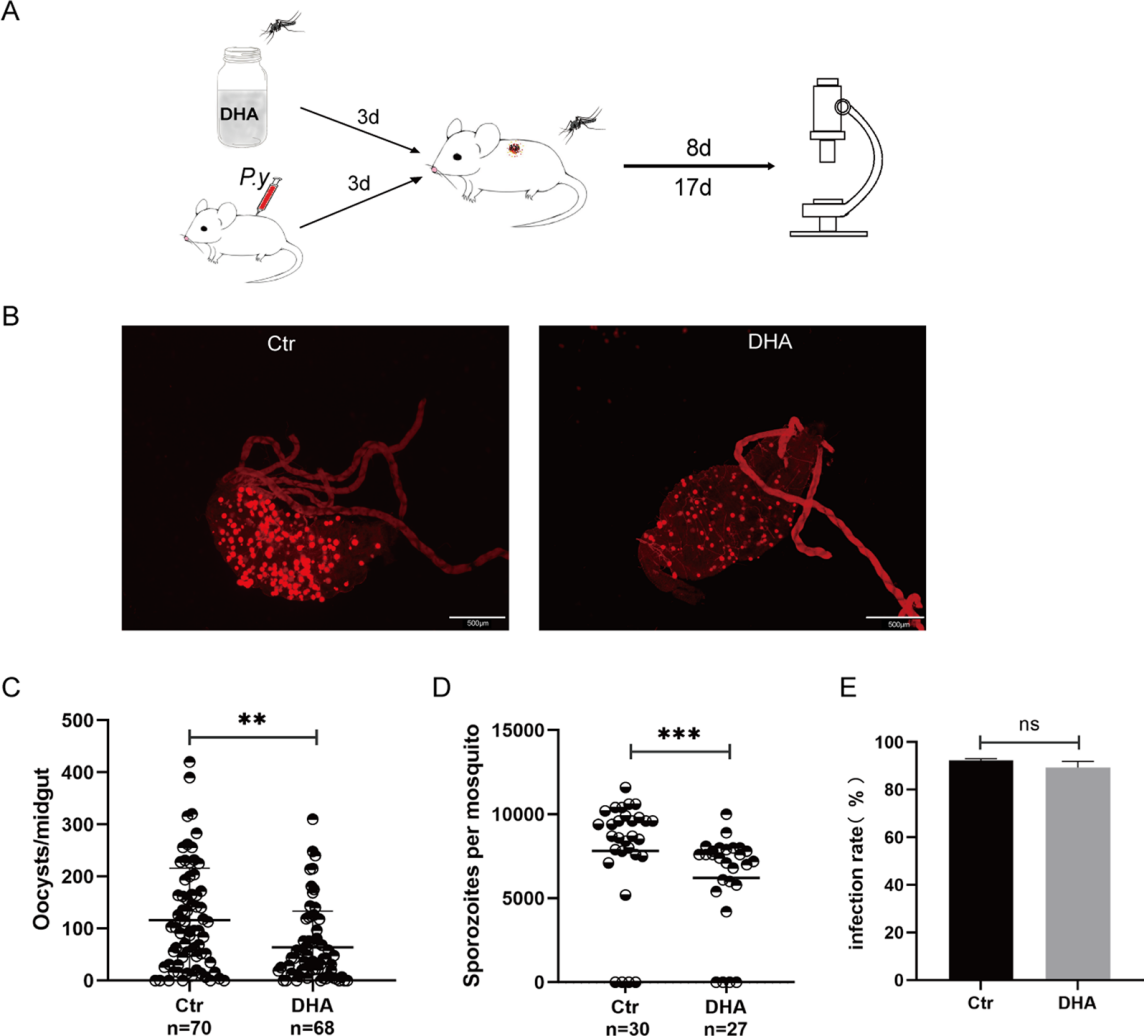
Feeding *A. stephensi* with DHA containing sugar inhibited *P. yoelii* oocyst development. **A** Schematic overview of DHA treatment and *A. stephensi* infection by *P. yoelii*. **B** The oocysts of *P. yoelii* in *A. stephensi*. **C**, **D** The infection densities of oocysts and sporozoites in mosquitoes between the DHA and control groups. **E** The oocyst infection rates of the DHA and control groups. Significance was determined by Mann–Whitney tests in **C** and **D** and by a chi-square test in **E**; **P < 0.01; ***P < 0.001; ns, no significant difference

To eliminate the influence of DHA metabolites on parasite development in the mosquitoes, *A. stephensi* were challenged with *P. yoelii* after feeding them a sugar solution containing DHA or DMSO for 3 consecutive days. Engorged females were dissected 8 and 17 days after infection and the oocysts and sporozoites were counted (Fig. 3A). Infection rates and densities of oocysts and sporozoites were compared between the two groups. The results showed that the infection densities in the DHA group were significantly lower than that in the control group (Fig. 3B–D). There was no significant difference in the infection rates of oocysts between the control group and the DHA treatment group (*χ*^2^ = 0.523, *df* = 1,* P* = 0.469) (Fig. 3E). These results indicate that direct feeding of DHA inhibited the development of malaria parasites in mosquitoes, which might decrease the malarial vector competence of *A. stephensi*.

### Toll signaling pathway was essential in DHA-mediated inhibition of* P. yoelii* development in* A. stephensi*

**Fig. 4:**
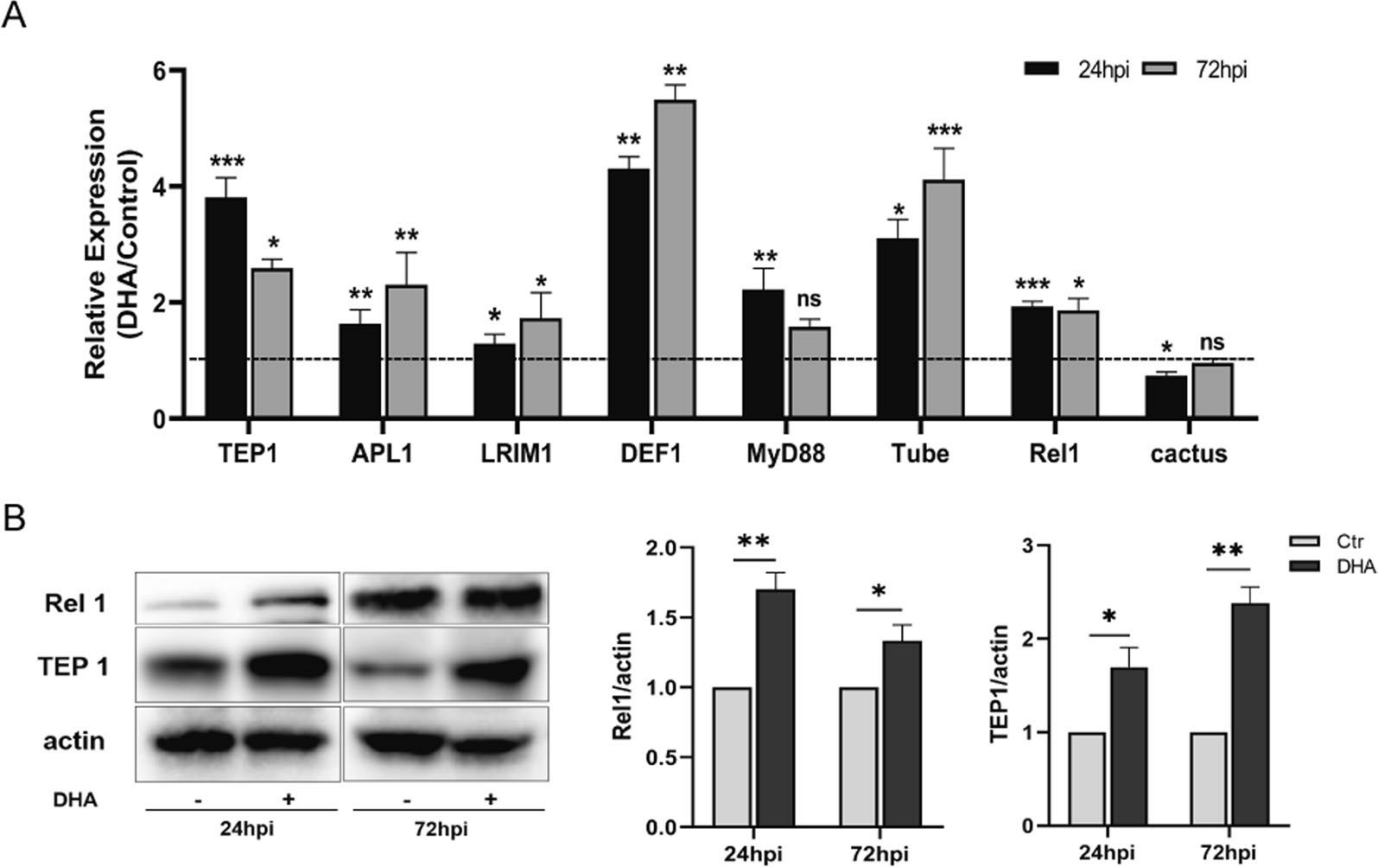
The impact of DHA treatment on the immune response of *A. stephensi* to *Plasmodium* infection. **A** The folds of transcriptional levels of TEP1, APL1, LRIM1, defensin 1, and immune genes of the Toll signaling pathway at 24 hpi and 72 hpi (DHA/control). The expression levels of targeted genes were normalized to S7. **B** Western blot analysis of Rel1 and TEP1 collected from the DHA-treated mosquitoes and the controls. Data from three independent western blots were statistically analyzed. Significance was determined by a Student’s *t*-test; *P < 0.05; **P < 0.01; ***P < 0.001; ns, no significant difference

Thioester-containing protein 1 (TEP1) plays a key role in killing *Plasmodium* parasites by forming complexes with the complement system of leucine-rich repeat proteins (LRIM1 and APL1) [26]. To understand whether these three molecules are involved in the inhibitory effect of DHA on *Plasmodium* development in *Anopheles*, the transcriptional levels of TEP1, LRIM, and APL1 were measured. The results showed that *TEP1*, LRIM1, and APL1C were significantly increased by DHA treatment at 24 and 72 h post infection (hpi) compared with those in the control group (Fig. 4A). As the expression of TEP1 can be regulated by the Toll signaling pathway [27], some key molecules of the Toll signaling pathway were detected using qPCR. The results showed that DHA treatment triggered the upregulation of myeloid differentiation primary-response gene 88 (*MyD88*), *Tube*, and the NF-κB transcription factor *Rel1* at 24 or 72 hpi and inhibited factor *Cactus* downregulation at 24 hpi compared with the control group (Fig. 4A). These results suggest that the Toll signaling pathway was activated in DHA-treated mosquitoes. Activation of the Toll pathway allows Rel1 to enter the nucleus and induces the expression of antimicrobial peptide (AMP) genes such as *DEF1* [28, 29]. To further confirm that DHA can activate the Toll signaling pathway, the transcriptional level of DEF1 was determined using qPCR. The expression of *DEF1* was upregulated by DHA treatment at 24 and 72 hpi compared with that in the control group (Fig. 4A). To better reflect the expression levels, western blotting was conducted to detect the proteins TEP1 and Rel1 in mosquitoes. Both proteins were upregulated by DHA treatment at 24 and 72 hpi compared with those in the control group (Fig. 4B). Overall, these results indicate that DHA treatment could induce high expression of TEP1 and AMP by activating the Toll signaling pathway, effectively impairing oocyst development and inhibiting the susceptibility of *A. stephensi* to *P. yoelii*.

### Vector competence of *A. stephensi* was recovered by interference of Rel1 expression

**Fig. 5:**
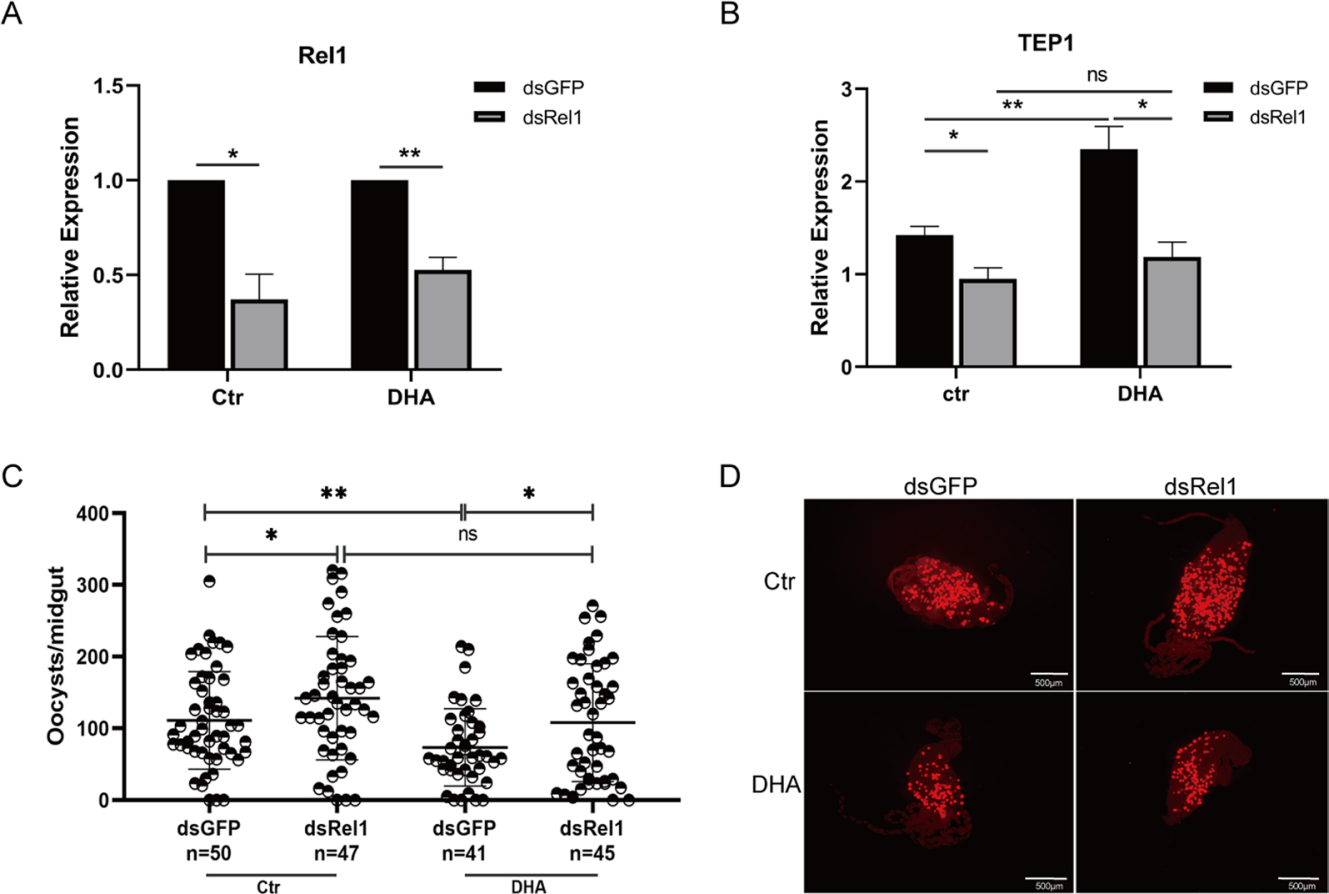
Silencing expression of Rel1 changed *P. yoelii* oocyst density in DHA-treated and untreated mosquitoes. **A** The silencing efficiency of Rel 1 in mosquitoes. **B** Influence of Rel 1 knockdown on gene expression of TEP1 in mosquitoes. **C** Influence of Rel 1 expression silencing on oocysts development in DHA-treated mosquitoes. **D** Oocysts shown on the midguts of mosquitoes under a fluorescence microscope. Significance was determined by Student’s *t*-test in **A** and **B**, and by Mann–Whitney tests in **C**; *P < 0.05; **P < 0.01; ns, no significant difference

To confirm the role of the Toll signaling pathway in the effect of DHA treatment on *P. yollii* development in *Anopheles*, the expression of Rel1, a key molecule in the Toll signaling pathway, was silenced using RNAi. The knockdown efficiency of Rel1 and expression levels of TEP1 were examined by qPCR. The silencing efficiency of Rel1 was 62.8% and 47.4% in the control and DHA-treated groups, respectively (Fig. 5A). Meanwhile, DHA treatment led to a significant increase in the expression of TEP1 compared to the untreated group in dsGFP mosquitoes, whereas the increase was eliminated when Rel1 was silenced by RNAi (Fig. 5B). These results indicate that changes in TEP1 transcription levels induced by DHA are regulated by Rel1. To further ascertain the role of Rel1 in the DHA-induced anti-*Plasmodium* defense against *P. yoelii* infection, we measured oocyst development after Rel1 RNAi in *A. stephensi*. Oocyst counts were compared between the dsRel1 and dsGFP groups in the presence or absence of DHA. There were more oocysts in the dsRel1 group than in the dsGFP group, whereas the inhibitory effect of DHA on oocyst development disappeared when Rel1 was knocked down by RNAi (Fig. 5C, D). In summary, these results indicate that the activation of the Toll signaling pathway by DHA significantly enhances the defense of *A. stephensi* against malaria parasites.

## Discussion

Artemisinin have been successful in controlling malaria, and its derivatives artemether, artesunate, and DHA are commonly used to treat *P. falciparum* malaria [30]. Artemisinins also have remarkable biological activities, including antifungal, antibacterial, antiviral, and anticancer properties [31]. Following entry into humans, artemisinin is first oxidized by cytochrome P450 to DHA in the liver and then enters the blood. Considerable efforts have been devoted to research on the disposal process of DHA, and a series of metabolites have been identified in the body [32, 33]. However, the effects of DHA and its metabolites on mosquitoes and mosquito pathogens remain unclear. When mosquitoes feed on the blood of people who have taken artemisinin, DHA or its metabolites can enter the body of mosquitoes and further affect their biological characteristics, such as the immune response to malaria parasite infection.

Our study showed that DHA effectively inhibited *Plasmodium* development in *Anopheles* mosquitoes. The number of oocysts and sporozoites in *Anopheles* was significantly reduced either by orally administration of DHA in mice or by DHA treatment via in vitro blood feeding and direct feeding to exclude the effect of DHA on the gametocytes in mice and the influence of DHA metabolites. The results indicated that DHA treatment significantly reduced the susceptibility of *Anopheles* mosquitoes to malaria parasites. This study provides an experimental basis for the application of artemisinin to block malarial transmission.

Mosquito’s innate immune system is the main line of defense against parasites and functions at multiple stages of *Plasmodium* infection [34]. There are three major signaling pathways involved in the defense against *Plasmodium*: the Toll, immune deficiency, and Janus kinase-signal transducers and activators of transcription (JAK-STAT) pathways [28]. We found that the expression of the Toll pathway genes, *MyD88*, *Tube*, and *Rel1*, was upregulated after DHA treatment at 24 and 72 hpi. The role of the Toll signaling pathway in the effect of DHA treatment on the susceptibility of *Anopheles* to *Plasmodium* was confirmed by RNAi. Therefore, the data strongly support the idea that DHA treatment boosts the Toll signaling pathway and thus decreases the vector competence of *A. stephensi* to *P. yoelii*. To investigate whether the effect of DHA on Toll pathway activation occurs in the absence of the parasite, the expression of TEP1 and the immune molecules in Toll pathway was detected using qRT–PCR. It was shown that DHA treatment had minimal effect on activation of the Toll pathway in uninfected mosquitoes (Supplementary Fig. 1). Besides, the other two innate immune pathways (immune deficiency and JAK-STAT) were assessed by analyzing their related gene expression. The gene expression of these two pathways changed irregularly following DHA treatment. Therefore, further research is needed to determine whether the other two signaling pathways are involved in the influence of DHA on the susceptibility of *Anopheles* to malaria parasites.

Although this study confirmed the inhibitory effect of DHA on *Plasmodium* development in *Anopheles* after excluding the effect of DHA on gametocytes in mice using in vitro blood feeding, it does not rule out the possibility that the influence of DHA on gametocytes in mice also affects the susceptibility of *Anopheles* to *Plasmodium*. Previous research has shown that artemisinin exhibits gametocidal activity against the early stages of *P. falciparum* gametocytes in vitro [35]. ACTs are known to be highly effective on the asexual stages of the malaria parasite but have little killing effect on mature male and female gametocytes [36, 37]. Even the mosquitoes were fed on blood of mice immediately after DHA was added to shorten the time for DHA acting on gametocytes, it cannot rule out the possibility of parasite death before mosquito infection. Besides, another scientific issue worth exploring is the potential impact of DHA on the differentiation and fertilization processes of *Plasmodium* in *Anopheles* and the following development such as ookinetes formation. It should also be considered that the observed effects may be due to the toxic properties of DHA directly.

Studies have shown that parasites develop resistance to artemisinin. The overuse of antimalarials in malarial areas puts immense drug selection pressure on *Plasmodium* to evolve resistance [30]. Artemisinin resistance was discovered in several countries in Southeast Asia in 2008 [38, 39]. Parasites exhibited signs of resistance to artemisinins in parts of Africa [40–42]. This selective advantage for resistance transmission could favor the acquisition of additional host specificities or polymorphisms. A resistant parasite isolate may be more likely to survive ACT treatment and its gametocytes may be more likely to be transmitted to mosquitoes [43]. The susceptibility of *Anopheles* mosquitoes to gametocytes from resistant strains is a scientific issue worthy of attention.

It is also worth investigating whether DHA metabolites affect the defense of *Anopheles* against invading malaria parasites or directly affect *Plasmodium* development in mosquitoes. Previously, owing to their low concentrations and complex detection backgrounds, DHA metabolites have been difficult to analyze in depth. Recently, DHA metabolites in blood were identified by ultraperformance liquid chromatography-electrospray ionization-quadrupole time-of-flight mass spectrometry [44], which is promising to detect the distribution of DHA and its metabolites in mosquito tissues. Understanding the dissemination of DHA and its metabolites in mosquito tissues is helpful to further investigate mechanisms of the inhibitory effect. The results of the present study showed that DHA reduced the susceptibility of *Anopheles* to malaria parasites in the absence of DHA metabolites, which does not exclude the possibility that DHA metabolites may affect the development of malaria parasites in *Anopheles*.

In addition, the effect of artemisinin on the gut microbiota of *Anopheles* mosquitoes may affect malaria transmission capacity, which is also a scientific issue worth exploring. Recent studies have suggested a complex triangular relationship between the gut flora, mosquitoes, and *Plasmodium*. The gut flora plays an important role in maintaining malarial vector competence of *Anopheles* [45, 46]. DHA has been reported to alter gut microbiota in mice [24]. However, the effects of DHA on mosquito gut microbiota remain poorly understood. Gut microflora-related mechanisms should be studied further in the future.

## Conclusions

This study focuses on the effect of DHA on susceptibility of *A. stephensi* to *P. yoelii* and the underlying mechanism. DHA treatment via different approaches significantly reduced oocyst and sporozoite counts in mosquitoes and increased the expression of key molecules involved in the Toll signaling pathway. When the Toll signaling pathway was blocked by silencing the expression of Rel1 using RNAi, the differences in oocyst counts disappeared. These results indicate that DHA inhibits the susceptibility of *A. stephensi* to *P. yoelii* by upregulating the Toll signaling pathway. This study provides a theoretical basis for the development of new agents that can block malaria transmission by modulating the immune response of *Anopheles* mosquitoes.

## Supplementary Information

Additional file 1: Table S1. The primers used in the PCR amplification.

Additional file 2: Fig. S1 The impact of DHA treatment on the immune response of *A. stephensi* without *Plasmodium* infection. The folds of transcriptional levels of TEP1, MyD88 and Rel1 at 24 hpi and 72 hpi (DHA/Control). The expression levels of targeted genes were normalized to S7. Significance was determined by Student’s *t*-test; *, *P* < 0.05.

## Data Availability

No datasets were generated or analyzed during the current study.

## Abbreviations

ACT: Artemisinin-based combination therapy
*A. stephensi*: *Anopheles stephensi*
Ctr: Control
DHA: Dihydroartemisinin
LRIM1: Leucine-rich repeat immune protein 1
*P. yoelii*: *Plasmodium yoelii*
TEP1: Thioester-containing protein 1
WHO: World Health Organization
