# Supplementary material for: Dihydroartemisinin suppresses the susceptibility of Anopheles stephensi to Plasmodium yoelii by activating the Toll signaling pathway
Source: Parasit Vectors. 2024 Oct 4;17:414. doi: 10.1186/s13071-024-06497-x (PMC11451267; doi:10.1186/s13071-024-06497-x)
Supplement: Supplementary file 1 — Additional file 1: Table S1. The primers used in the PCR amplification. [file 13071_2024_6497_MOESM1_ESM.docx]

Table S1. Primers used for PCR amplification.

| Target gene | Primer sequence (5′ to 3′) |
| --- | --- |
| *S7* | F: CTAACGACACGAAGACCACAAGA |
|  | R: CAACCTGCAACGACAGCAAAA |
| *T7-dsGFP* | F: TAATACGACTCACTATAGGGAGTCAAGTTCAACGTGTCCGGCG |
|  | R: TAATACGACTCACTATAGGGAGAGGACCATTTGATCGCGCTT |
| *TEP 1* | F: ACCGATTGTCCAAGTTCTCG |
|  | R: AGCGCATCTGGTTCTGGTAG |
| *APL1* | F: AGAGTCGGCAGGCGTTCAA |
|  | R: GCTTGTCGGTCTTCAGGGTCAG |
| *LRIM1* | F: AGCTCGTGCTGTTGAGTGAA |
|  | R: CGTACGGTCGCAATCGAAAC |
| *MyD88* | F: TCGGCGGACAGTGACATTATTACG |
|  | R: TCACGATCCTTCAGACACAGTTGC |
| *Tube* | F: CGATTCCAGCAGCACAGACTC |
|  | R: GTCGGTAGCGTTACACAGCAG |
| *Rel 1* | F: GAACTGGATTCGGTCACGCTAAGG |
|  | R: CGGCAGATAATCAGGTCGGACATG |
| *T7-dsRel1* | F: TAATACGACTCACTATAGGGAGTCGGGCTGAAGGCGTTGACC |
|  | R: TAATACGACTCACTATAGGGAGTGCCACCTTGAATCGTCTGA |
| *Cactus* | F: CGCTTGCAGATGCTAGTGGTCAG |
|  | R: CCGCTGTTCGCTGGCTGTTC |
| *DEF1* | F: AACGCTCATCTGTGCCGTAG |
|  | R: CGTCTCTTCGGGCAGTTCAT |

T7-Primers for dsRNA synthesis, the other primers for real time quantitative PCR.
