# Supplementary figures and images for: Dihydroartemisinin suppresses the susceptibility of Anopheles stephensi to Plasmodium yoelii by activating the Toll signaling pathway
Source: Parasit Vectors. 2024 Oct 4;17:414. doi: 10.1186/s13071-024-06497-x (PMC11451267; doi:10.1186/s13071-024-06497-x)

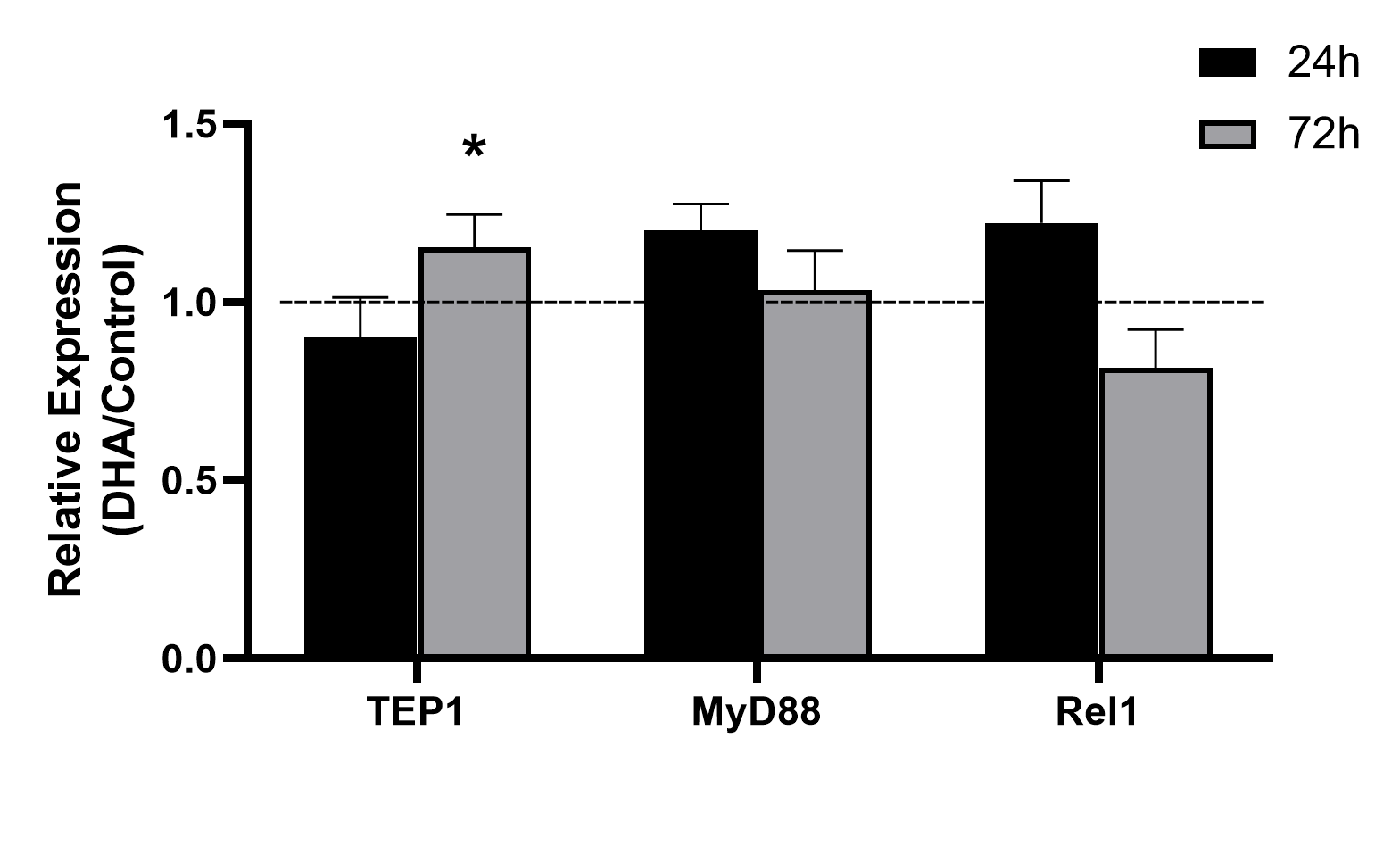

Supplement: Supplementary file 2 — Additional file 2: Fig. S1 The impact of DHA treatment on the immune response of A. stephensi without Plasmodium infection. The folds of transcriptional levels of TEP1, MyD88 and Rel1 at 24 hpi and 72 hpi (DHA/Control). The expression levels of targeted genes were normalized to S7. Significance was determined by Student’s t-test; *, P < 0.05. [file 13071_2024_6497_MOESM2_ESM.tif]
